# Preliminary Clinical Investigation of Combinatorial Pharmacogenomic Testing for the Optimized Treatment of Depression: A Randomized Single-Blind Study

**DOI:** 10.3389/fnins.2019.00960

**Published:** 2019-09-13

**Authors:** Xiaoxiao Shan, Wenli Zhao, Yan Qiu, Haishan Wu, Jindong Chen, Yiru Fang, Wenbin Guo, Lehua Li

**Affiliations:** ^1^Department of Psychiatry, The Second Xiangya Hospital, Central South University, Changsha, China; ^2^National Clinical Research Center on Mental Disorders, Changsha, China; ^3^Division of Mood Disorders, Shanghai Mental Health Center, Shanghai Jiao Tong University School of Medicine, Shanghai, China

**Keywords:** depression, antidepressant drugs, pharmacogenomics testing, clinical efficiency, genes

## Abstract

This study aims to explore the potential benefits of antidepressant drugs related to metabolic enzyme and drug-targeted genes, identify the optimal treatment of major depression, and provide a reference for individualized medication selection. A prospective randomized single-blind investigation was conducted for 8 weeks. A pharmacogenomic-based interpretive report was provided to the treating physician in the guided group. Patients in this group were informed that their medication selection was directed by DNA testing. In the unguided group, treatment was provided based on the clinical experience of the physician without the guidance of pharmacogenomic testing. Pharmacogenomic-based interpretive report was not provided to these patients until treatment completion. The 17-item Hamilton depression scale (HAMD-17), Hamilton anxiety scale, and treatment emergent symptom scale were used to assess the clinical efficacy and side effects at baseline and after 2, 4, and 8 weeks of treatment. Among the 80 initially enrolled patients with depression, 71 participated in the full data analysis sets and were designated into guided (31) and unguided (40) groups, respectively. No significant difference (*P* > 0.05) in HAMD-17 total scores, response and remission rates was found between the guided and unguided groups at the end of the treatment. The incidence rate of adverse reaction was 55.56% in guided group and 57.89% in the unguided group. Our study suggested that pharmacogenomic testing might not considerably improve the clinical efficiency and safety for the guided group.

## Introduction

Major depressive disorder (MDD) is a common mental illness with high incidence and recurrence rate, which increases the risk of committing suicide and brings heavy economic burden to society. Along with AIDS and ischemic heart disease, depression is projected to be one of the leading causes of disease burdens worldwide by 2030 (Mathers and Loncar, 2006).

Antidepressant drugs are the main therapy for depression. Appropriate and timely therapy can improve clinical remission rate and reduce disease burden. According to the Sequenced Treatment Alternatives to Relieve Depression (STAR^∗^D) study, approximately one-third of patients with MDD would respond to the first guideline-notified antidepressant (Trivedi et al., 2006; Gaynes et al., 2009). The other one-third fails to acquire remission from their acute episode after multiple trials with mechanistically dissimilar antidepressants. Patients’ response to psychotropic drugs, side effects, and doses of antidepressant drugs considerably varies depending on many factors, such as age, gender, diagnostic accuracy, potential drug interaction, nutritional status, genetics, and patient compliance. Genetic variation is one of the important reasons for the differences in inter-individual efficacy. The variance in antidepressant responses is approximately 42% through common genetic variation (Tansey et al., 2013).

Pharmacogenomic testing is a promising method for the selection of antidepressants and has been developed to offer a priori prediction on the kind of medication that might produce the lowest risk of adverse events and/or the highest likelihood of treatment response for a specific individual (Singh et al., 2017). Pharmacogenomics mainly studies the effects of gene polymorphisms on the variability of pharmacodynamics (involving the evaluation of receptor and transporter function about the drug mechanism of action) and pharmacokinetics [involving the evaluation of cytochrome P450 (CYP) enzyme activity about the drug metabolism], and is expected to improve clinical effects and reduce adverse reactions for patients (Singh et al., 2017). P450 enzymes (mainly CYP2D6 and CYP2C19), serotonin transporter gene (SLC6A4), serotonin receptor gene (HTR2C, HTR2A), and p-glycoprotein drug transporter (ABCB1) gene, which encodes p-glycoprotein and plays an important role in drug bioavailability and response to drugs, may all be associated with drug metabolism, safety, and tolerance (Altar et al., 2013; Breitenstein et al., 2014; Fabbri and Serretti, 2015; Outhred et al., 2016).

The CYP enzyme superfamily is involved in the oxidation and reduction of xenobiotic and endogenous substances. CYP2D6 is the major enzyme responsible for the metabolism of fluoxetine, fluvoxamine, and paroxetine, and it contributes in sertraline metabolism. CYP2C19 is mainly involved in the metabolism of escitalopram, citalopram, and sertraline (Probst-Schendzielorz et al., 2015). These isoenzyme genes are highly polymorphic, in which alleles might present normal, partially, or totally defective activity. The polymorphisms of CYP2D6 and CYP2C19 affect enzyme activity in different ways: no enzyme activity resulting from two defective alleles coding for the metabolic isoenzyme (poor metabolizer, PM); decreased activity because of one defective and one functional allele (intermediate metabolizer, IM); and normal activity because of two functional alleles (extensive metabolizer, EM) related to gene duplication (ultrarapid metabolizer, UM) (Bertilsson et al., 1997; Griese et al., 1998). These different phenotypes may cause varying results. For instance, inefficacy or therapeutic resistance occurs because the plasma levels of drugs are reduced by the polymorphic metabolizing enzymes in UM phenotypes, whereas slow metabolism (PM) might increase plasma levels and toxicity. Jukic et al. reported that CYP2C19 genotype has a substantial effect on the exposure and therapeutic failure of escitalopram. They found that, compared with those in the extensive metabolizers’ group (CYP2C19^∗^1/^∗^1), escitalopram serum concentrations were significantly increased 3.3-fold in the poor metabolizers’ group (CYP2C19^∗^2/^∗^2) and significantly decreased by 20% in the ultrarapid metabolizers’ group (CYP1C19^∗^17/^∗^17). This finding suggests the underlying clinical utility of CYP2C19 genotyping for the individualization of therapy (Jukic et al., 2018). A previous meta-analysis also reported that dose recommendations based on CYP2D6 or CYP2C19 may contribute to individualized drug therapy (Kirchheiner et al., 2010).

Medications have been increasingly incorporated with different pharmacogenomic biomarkers for the poor metabolizers of CYP2D6 and CYP2C19 enzymes and dosage warnings on drug labels. For example, patients with poor metabolizers for the CYP2D6 enzyme should receive the recommended starting dose of fluvoxamine at 70 or 50% of the normal dose. Moreover, the Clinical Pharmacogenetics Implementation Consortium (CPIC) published the guidelines for psychotropic drug selection, as well as dose adjustment of selective serotonin reuptake inhibitors and tricyclic antidepressants based on CYP2D6 and CYP2C19 genotypes (Hicks et al., 2013, 2015). The United States and EU have incorporated pharmacogenomic information into drug labels. The Food and Drug Administration (FDA) has already included the related labeling information of genetic testing for more than 100 medications, among which at least 9 antidepressants have been included (Frueh et al., 2012).

Although multiple methods of testing have been developed, pharmacogenomic testing normally involves the assessment of one or more pharmacodynamic (mechanism of action) and pharmacokinetic (drug metabolism) genes. The extent of gene–drug interactions is assessed based on a single gene (single gene testing) or on multiple genes (multigene testing) to recognize medications that are potentially unsafe and ineffective. Altar et al. found that the combinatorial multigene pharmacogenomic testing contributes to the selection of genetically proper medications and the prediction of clinical outcomes for depressed patients; multigene testing provides greater healthcare utilizations than phenotypes based on single genes (Altar et al., 2015b). Combinatorial multigene pharmacogenomic testing is the approach of integrating multiple genetic factors, including pharmacodynamic genes predicting therapeutic response and side effects, and pharmacokinetic genes predicting medication exposure and appropriate dosing, to identify individuals with gene–drug interactions and predict potential clinical outcomes, such as the possibility of response to treatment. The combinatorial-multiple genetic approach has showed clinical validity and utility based on pharmacogenomic platform. Combinatorial pharmacogenomic testing may improve the clinical outcomes of patients with MDD (Hall-Flavin et al., 2012, 2013; Perez et al., 2017; Tanner et al., 2018), while reducing polypharmacy and healthcare costs (Brown et al., 2017), mainly by utilizing GeneSight testing platform (AssureRx Health, Inc., United States). This method is a commercially available combinatorial pharmacogenomic test that analyzes cheek swab tissue and generates a report to aid prescription selection based on the pharmacodynamics and pharmacokinetic profile of an individual.

Other combinatorial pharmacogenomic tests from different platforms have also been reported. Brennan et al. (2015) utilized the Genecept assay (Genomind, Pennsylvania), a combinatorial pharmacogenomic testing method that similarly guides prescribers based on pharmacodynamics and pharmacokinetics. Singh utilized CNSDose testing, a combinatorial pharmacogenomic test that only evaluates genes involved in pharmacokinetics to assist in medication dosing (Singh, 2015). Combinatorial pharmacogenomic testing uniquely explains multiple metabolic pathways and mechanisms for each medication, thereby contributing to a greater predictive power for patient outcomes than single gene testing (Altar et al., 2015b). However, the combinatorial pharmacogenomic testing of psychotropic drugs has not been widely used in clinical practice. Evidence supporting pharmacogenomic testing for antidepressant responses in clinical application is still lacking. Thus, improving the usage of pharmacogenomics in treatments remains a challenge.

This study aims to explore the potential benefit of pharmacogenomic testing for the optimal treatment of MDD. Eighty patients with MDD were recruited to explore the effect of pharmacogenomic testing on clinical outcome. The clinical status of patients with MDD was obtained at four time points (baseline, 2, 4 and 8 weeks of treatment). Basing on the abovementioned studies, we hypothesized that pharmacogenomic testing-guided treatment may remarkably improve clinical efficiency and safety for patients with MDD.

## Materials and Methods

### Participants

Eighty patients with MDD (outpatients and inpatients) were initially enrolled from the Department of Psychiatry of the Second Xiangya Hospital, Central South University in China, from September 2017 to July 2018. Depressive disorder was diagnosed using the Structural Clinical Interview for Diagnostic and Statistical Manual of Mental Disorders, fifth edition (DSM-5). The patients were 18–51 years old and had at least a junior high school education level with the ability to understand survey contents. They were randomly allocated to guided and unguided groups. All enrolled patients are members of the most common Han population in China. Inclusion criteria were as follows: (1) subjects with a 17-item Hamilton depression scale (HAMD-17) total scores of ≥ 17 at baseline and the first item of the HAM D-17 (depressive mood) ≥ 2; (2) never received psychiatric treatment or have interrupted antidepressant medication for more than 2 weeks (fluoxetine for at least 4 weeks); and (3) with no psychotic symptoms. Exclusion criteria for all participants were as follows: having any other psychiatric diagnoses according to DSM-5; any physical illness such as liver and kidney diseases, cardiovascular diseases; any combination with other antipsychotic medications (both low and high doses), including typical and atypical antipsychotic and mood stabilizer; and pregnancy.

### Study Description

This prospective randomized single-blind study aimed to assess the effectiveness pharmacogenetic testing for medication therapy selection in patients with depression. Subjects meeting all of the inclusion criteria and none of the exclusion criteria were enrolled and randomly categorized into guided or unguided group according to a random number list. Both groups were treated by the same clinical psychiatrist, who was responsible for choosing drug and dosing schedule best-fitted to each patient. The assessment scales were conducted by another clinical psychiatrist who was blinded regarding patient allocation. For the patients in the guided group, DNA was gathered by buccal swab at baseline. Within 48 h of sample collection, the pharmacogenomic-based report was offered to the treating psychiatrist. These patients were notified that their drug selection was guided by the DNA testing. For the patients in the unguided group, DNA was gathered by buccal swab at the onset of treatment, and pharmacogenomic-based report was created but not provided to the physician until the completion of 8 weeks of treatment. Hence, these patients received antidepressant treatment according to the psychiatrist clinical experience without the aid of pharmacogenomic testing. The drug dosage was gradually increased to the effective dose on the basis of the patient’s condition. Each patient participated in a single group. Except for the pharmacogenomic testing and the interpretive report, no extra pharmacogenomic education was offered to the participants or physicians in the guided group. The pharmacogenomic testing was offered free of charge to the study participants, and no extra incentive was provided to them. The genetic testing samples were oral mucosal exfoliated cells obtained through disposable buccal swabs by a psychiatrist at baseline following the manufacturer’s instructions. The buccal samples were then transported from the hospital to the laboratory in Conlight Medical Institute in Shanghai on the same day.

The HAMD-17 was used to evaluate symptomatic severity at baseline and after treatment. The following formula was applied to calculate the reduction ratio (RR) of the HAMD-17 total scores and evaluate treatment effect: RR = (HAMD-17_total___1_-HAMD-17_total___2_)/HAMD-17_total___1_. HAMD-17_total___1_ refers to the HAMD-17 total scores at baseline, whereas HAMD-17_total___2_ is the HAMD-17 total scores after 8 weeks of treatment. Response to therapy was defined as the RR of HAMD-17 by ≥ 50%. Remission was defined as the final HAMD-17 score ≤ 7 at the end of the 8-week treatment. Blood routine, liver function, and renal function tests were conducted to assess the physical condition of patients at baseline and at 8th week. Hamilton anxiety scale (HAMA) was used to evaluate anxiety symptoms. Treatment emergent symptom scale (TESS) was employed to assess the treatment side effects (Lyerly and Abbott, 1973).

The Ethics Committee of the Second Xiangya Hospital of Central South University approved this study. After receiving a complete explanation, all participants submitted their written informed consent.

### Genotyping Procedure

Genomic DNA was isolated from the buccal samples and then transported to Conlight Medical Institute in Shanghai and analyzed using TaqMan probe–PCR and mass array. Polymerase chain reaction (PCR) was applied to amplify the relevant genomic regions. Genotyping of single nucleotide polymorphisms was conducted by ABI 7500 real-time fluorescence quantitative PCR combined with TaqMan probe and arms-PCR. MassARRAY DNA based on Matrix Assisted Laser Desorption/Ionization-Time of Flight Mass Spectrometry was employed to accurately identify mutation type. Genetic polymorphisms including CYP2C19, CYP2D6, CYP1A2, SLC6A4, and 5-HTR2A, that affect the response of most antidepressant medications, were detected. Gene loci were identified through FDA specifications, CPIC official guidelines, clinical trial data, authoritative literature, and relevant genome databases. The candidate gene loci are all based on the characteristics and distribution frequency of Asians especially Chinese people. The CYP2D6 alleles identified were: ^∗^1,^∗^2, ^∗^2A, ^∗^3, ^∗^4, ^∗^5, ^∗^6, ^∗^7, ^∗^8, ^∗^9, ^∗^10, ^∗^11, ^∗^12, ^∗^14A, ^∗^14B, ^∗^15, ^∗^17, ^∗^36,^∗^41, and CNV. The identified CYP2C19 alleles were: ^∗^1, ^∗^2, ^∗^3, ^∗^4, ^∗^5, ^∗^6, ^∗^7, ^∗^8, ^∗^9, and ^∗^17. The identified CYP1A2 alleles were: ^∗^1, ^∗^1C, ^∗^1E, ^∗^1F, ^∗^1K, ^∗^3, ^∗^4, ^∗^6, ^∗^7, ^∗^8, ^∗^11, ^∗^15, and ^∗^16. The identified HTR2A alleles were single nucleotide polymorphism rs7997012, A > G. Long or short segments of the SLC6A4 promoter region were identified because of an insertion or missing base pairs.

**FIGURE 1:**
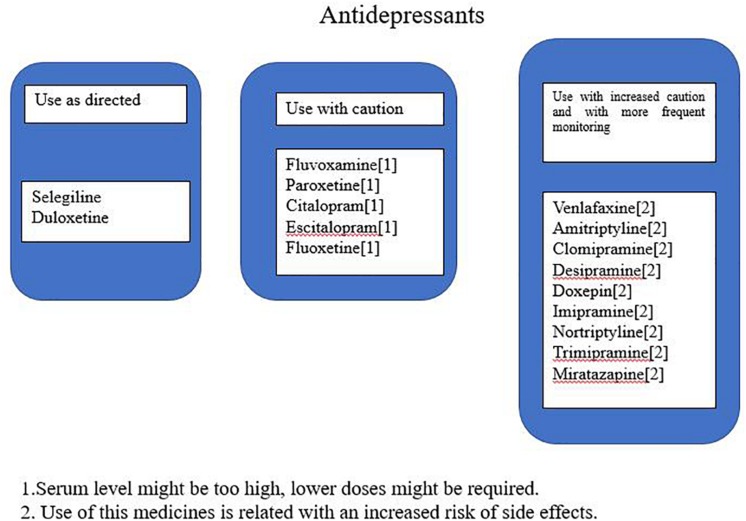
An example of a report for one patient.

The genotype results were used in a proprietary interpretive report, which incorporates genetic information with pharmacological profile for antidepressants in the panel. This algorithm is based on the genotyping of both copies of five selected genes either for their pharmacokinetic significance in the metabolism of majority antidepressants or because of differences in treatment response based on pharmacodynamic considerations. These five genes include three pharmacokinetic genes (CYP2C19, CYP2D6, and CYP1A2) and two pharmacodynamic genes (SLC6A4 and 5-HTR2A). The drugs in the interpretive report were classified into three advisory categories: “use as directed,” “use with caution,” or “use with increased caution and more frequent monitoring” to improve the genotyping results clinical relevance for clinicians. Medications in the “use as directed” were predicted to be valid without any clinical modifications, those in the “use with caution” were predicted to be valid with dose modification, and those in the “use with increased caution and more frequent monitoring” were predicted to have severe gene–drug interactions that may substantially affect the medication’s safety and/or efficacy (Figure 1 shows an example of one patient report). Footnotes related to each drug in the “use with caution” or “use with increased caution and more frequent monitoring” bins offer the detailed information of the gene–drug interactions and may state the following: the serum levels of the drugs are extremely high or low; the genotype implies low response or reduced efficacy (e.g., CYP450 UM or SLC6A4 S/S genotype); or the drug may increase potential adverse events (e.g., CYP450 PM phenotype).

### Statistical Analysis

Continuous variables between groups were analyzed using Wilcoxon test. Dichotomous or categorical variables between groups were analyzed using chi-square test. Repeated analyses of covariance were performed to compare the group differences in efficacy indicators with age and gender as covariances. Given the high dropout rate of participant, full analysis set (FAS) and per protocol set (PPS) were used to analyze the indexes for therapeutic effects, with FAS results as the main findings. All analyses were conducted using SSPS 20.0 software. PPS refers to the patients that have completed the entire research program, including follow-up visits, without major protocol deviation that might have affected the primary efficacy evaluation. FAS includes patients who have received at least one treatment and had related efficacy evaluation, but failed to complete the whole treatment process, and those who completed entire research program. The results of the last observation were carried forward when the main efficacy indicators were missing.

## Results

### Demographic Characteristics

**FIGURE 2:**
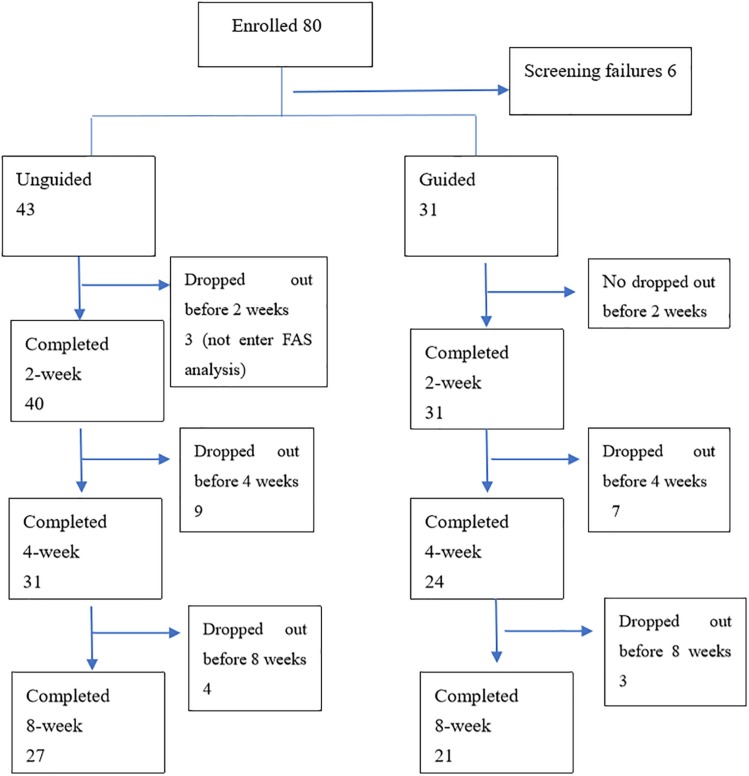
Chart of subject accrual and dropouts.

Eighty patients with MDD were enrolled in the study. Six patients failed the screening and were excluded (four patients had severe suicidal ideation and behavior, and two patients with HAMD-17 total score that were below 17). Three patients failed to receive at least one treatment follow-up and failed to have at least one efficacy evaluation. Hence, 71 patients entered FAS. Forty-eight patients completed all the follow-up and entered PPS. Among the 74 remaining patients, 71 (31 in the guided group and 40 in the unguided group) completed the 2nd week follow-up, 55 (24 in the guided group and 31 in the unguided group) completed the 4th week follow-up, and 48 (21 in the guided group and 27 in the unguided group) completed the 8th week follow-up (Figure 2). No differences in age (*Z* = −1.063, *P* = 0.288), sex (*F* = 0.104, *P* = 0.748), educational background (*F* = 0.455, *P* = 0.500), number of episodes (*F* = 2.655, *P* = 0.301), and duration of current episode (Z = −1.133, *P* = 0.257) were observed between the guided and unguided groups (*p* > 0.05). Table 1 presents the detailed information on the demographics of the participants. Table 2 displays the detailed information on the antidepressants used for treatment.

**TABLE 1:** Demographic characteristics of the study.

	**Guided *N* = 31**	**Unguided *N* = 40**	**F/Z**	***P*-value**
Age (years)	26.52 ± 7.92	28.85 ± 8.93	1.063	0.288^a^
Sex (Male/female)	12/19	14/26	0.104	0.748^b^
Education (senior high school/university)	10/21	10/30	0.455	0.500^b^
Duration of the current episode (months)	7.16 ± 2.81	6.43 ± 2.34	–1.133	0.257^a^
The number of episodes(1/2/3)	20/9/2	18/17/5	2.655	0.301^b^

*^*a*^The *p*-values were obtained by the Wilcoxon test. ^*b*^The *p*-values were obtained by the chi-square test.*

**TABLE 2:** The usage and dosage of antidepressants in patients of guided and unguided groups.

	**Guided group**	**(mg/d)**	**Unguided group**	**(mg/d)**
01	sertraline	150	mirtazapine	30
02	Duloxetine	60	sertraline	100
03	Paroxetine	40	Escitalopram	20
04	venlafaxine	150	Fluoxetine	40
05	sertraline	100	sertraline	50
06	mirtazapine	15	Duloxetine	60
07	sertraline	100	venlafaxine	150
08	Duloxetine	90	sertraline	100
09	Fluoxetine	40	Paroxetine	60
10	sertraline	100	Duloxetine	90
11	venlafaxine	150	sertraline	100
12	mirtazapine	30	venlafaxine	75
13	venlafaxine	150	mirtazapine	15
14	sertraline	150	Escitalopram	15
15	Paroxetine	40	Fluoxetine	40
16	Escitalopram	20	venlafaxine	150
17	Paroxetine	40	Bupropion	100
18	mirtazapine	30	Escitalopram	20
19	sertraline	100	Trazodone	150
20	Venlafaxine	150	Fluvoxamine	200
21	Duloxetine	90	Duloxetine	120
22			Trazodone	100
23			Paroxetine	20
24			sertraline	150
25			clomipramine	100
26			Paroxetine	40
27			mirtazapine	30

### Genotype Distribution

In the guided group, the CYP2D6 metabolic capacity phenotypes were 3.22% ultrarapid metabolizers, 48.39% extensive metabolizers, and 48.39% intermediate metabolizers. The CYP2C19 metabolic capacity phenotypes were 29.03% extensive metabolizers, 61.29% intermediate metabolizers, and 9.68% poor metabolizers. The CYP1A2 metabolic capacity phenotypes were 35.48% extensive metabolizers and 64.52% ultrarapid metabolizers. Their SLC6A4 genotypes were 6.45% L/L, 6.45% L/S, and 87.10% S/S. Their HTR2A rs7997012 genotypes were 3.23% A/A, 38.71% A/G, and 58.06% G/G.

In the unguided group, the CYP2D6 metabolic capacity phenotypes were 2.5% ultrarapid metabolizers, 55% extensive metabolizers, 37.5% intermediate metabolizers, and 5% poor metabolizers. The CYP2C19 metabolic capacity phenotypes were 45% extensive metabolizers, 47.5% intermediate metabolizers, and 7.5% poor metabolizers. The CYP1A2 metabolic capacity phenotypes were 37.5% extensive metabolizers and 62.5% ultrarapid metabolizers. Their SLC6A4 genotypes were 7.5% L/L, 30% L/S, and 62.5% S/S. Their HTR2A rs7997012 genotypes were 10% A/A, 30% A/G, and 60% G/G. The genotype distributions for the SLC6A4 phenotype differed between the two groups and showed no difference with the other four genes. Table 3 presents detailed information regarding the distribution of genotypes.

**TABLE 3:** Distribution of phenotypes in two groups.

**Gene phenotype**	**Guided (%)**	**Unguided (%)**	***P*-value**
CYP2D6			
Poor metabolizer	0	2 (5)	.
Intermediate metabolizer	15 (48.39)	15 (37.50)	0.637
Extensive metabolizer	15 (48.39)	22 (55)	
Ultrarapid metabolizer	1 (3.22)	1 (2.5)	
CYP2C19			
Poor metabolizer	3 (9.68)	3 (7.5)	0.370
Intermediate metabolizer	19 (61.29)	19 (47.5)	
Extensive metabolizer	9 (29.03)	18 (45)	
CYP1A2			
Extensive metabolizer	11 (35.48)	15 (37.5)	1.00
Ultrarapid metabolizer	20 (64.52)	25 (62.5)	
SLC6A4			
LL	2 (6.45)	3 (7.5)	
LS	2 (6.45)	12 (30)	0.036
SS	27 (87.10)	25 (62.5)	
HTR2A			
AA	1 (3.23)	4 (10)	0.519
AG	12 (38.71)	12 (30)	
GG	18 (58.06)	24 (60)	

*The *p*-values were obtained by the chi-square test.*

### Depression Outcomes

**FIGURE 3:**
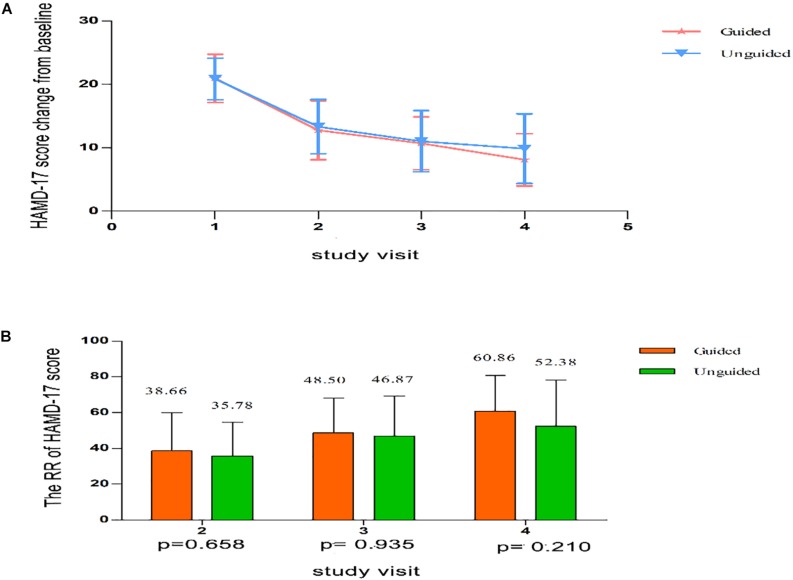
**(A)** HAMD-17 scores at each time point. Points represent mean scores for each group at each time point. Bars represent SD. **(B)** The RR of HAMD-17 score at each time point. Values above histogram bars represent group mean scores. Bars represent SD. *P*-values are derived using the Repeated analyses of covariance. HAMD-17 = 17-item Hamilton depression scale; RR, reduction ratio.

**FIGURE 4:**
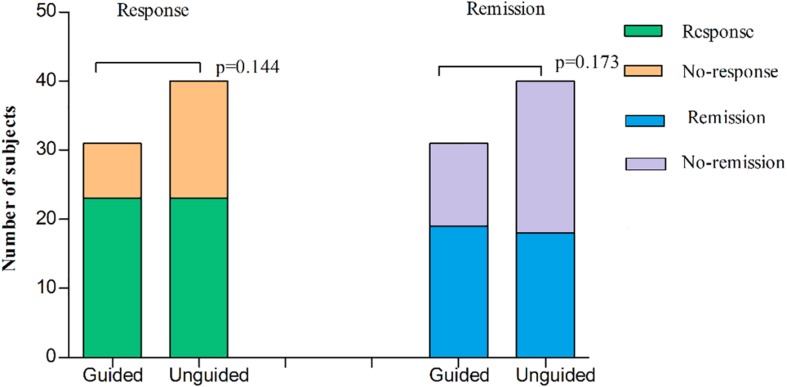
The subjects of response or remission at 8 weeks treatment in the guided group and the unguided group. Response to therapy was defined as the RR of HAMD-17 by ≥ 50%. Remission was defined as the final HAMD-17 score ≤ 7 at the end of the 8-week treatment. The two histogram bars on the left represent the treatment response of two groups and the two right represent treatment remission of two groups. *P*-values on the left and right represent the comparison of response rates and remission rates respectively, between two groups. *P*-values are derived using χ^2^ tests. HAMD-17 = 17-item Hamilton depression scale; RR, reduction ratio.

We found that the HAMD-17 total scores significantly decreased from baseline to 8 weeks within group (*P* < 0.01). However, no significant difference was found in the HAMD-17 total scores at each time point between the unguided and guided groups (Figure 3A). The RR of HAMD-17 scores at 8 weeks was 60.86% in the guided group and 52.38% in the unguided group with no significant difference (Figure 3B). After 8 weeks of treatment, the response rates of the guided and unguided groups were 74.19% (23/31) and 57.5% (23/40), respectively. The remission rates of the guided and unguided groups were 61.29% (19/31) and 45.0% (18/40), respectively (Figure 4). Although the response and remission rates were higher in the guided group than in the unguided group, both rates were not statistically significant. Tables 4, 5 present the detailed information of the analyses. Moreover, no significant difference in the HAMA total scores was observed at each time point between two groups (Table 6).

**TABLE 4:** Comparison of the HAMD scores between Guided and Unguided group at each time point.

	**FAS**				**PPS**			
	**Guided *N* = 31**	**Unguided *N* = 40**	***F***	***P*-value^a^**	**Guided *N* = 21**	**Unguided *N* = 27**	***F***	***P*-value^a^**
Baseline	20.97 ± 3.80	20.88 ± 3.28	0.003	0.958	21.24 ± 4.29	20.74 ± 3.39	0.077	0.782
2 Weeks	12.77 ± 4.67^∗^	13.33 ± 4.27^∗^	0.154	0.696	13.67 ± 4.34^∗^	12.74 ± 4.17^∗^	0.526	0.472
4 Weeks	10.68 ± 4.17^∗^	11.03 ± 4.83^∗^	0.016	0.901	10.57 ± 3.83^∗^	9.96 ± 4.25^∗^	0.523	0.473
8 Weeks	8.10 ± 4.12^∗^	9.88 ± 5.49^∗^	1.635	0.205	6.76 ± 2.88^∗^	8.26 ± 4.84^∗^	1.007	0.321

*^∗^Compared with the baseline of the same group *P* < 0.01. Values are represented as mean ± sd. *P*-values^*a*^ were obtained using the Repeated analyses of covariance.*

**TABLE 5:** Comparison of the response rates and remission rates between the Guided and Unguided group at the end of 8 weeks.

	**FAS**			**PPS**		
	**Guided *N* = 31**	**Unguided *N* = 40**	***P*-value**	**Guided *N* = 21**	**Unguided *N* = 27**	***P*-value**
Response rates%	23/31(74.19)	23/40(57.5)	0.144	19/21(90.48)	19/27(70.37)	0.152
Remission rates%	19/31(61.29)	18/40(45.0)	0.173	16/21(76.19)	14/27(51.85)	0.133

*The *p*-values were obtained by the chi-square test.*

**TABLE 6:** Comparison of the HAMA scores between Guided and Unguided group at each time point.

	**FAS**				**PPS**			
	**Guided *N* = 31**	**Unguided *N* = 40**	***F***	***P*-value^a^**	**Guided *N* = 21**	**Unguided *N* = 27**	***F***	***P*-value^a^**
Baseline	16.45 ± 6.45	16.13 ± 7.20	0.033	0.856	16.24 ± 6.36	14.74 ± 6.41	0.222	0.640
2 Weeks	9.65 ± 4.17^∗^	9.75 ± 4.78^∗^	0.0003	0.985	9.62 ± 4.16^∗^	8.56 ± 3.69^∗^	1.399	0.243
4 Weeks	8.13 ± 4.12^∗^	8.08 ± 4.91^∗^	0.020	0.889	7.38 ± 3.87^∗^	7.04 ± 4.42^∗^	0.039	0.844
8 Weeks	7.10 ± 3.76^∗^	7.28 ± 4.94^∗^	0.002	0.961	5.86 ± 2.74^∗^	5.85 ± 4.12^∗^	0.001	0.981

*^∗^Compared with the baseline of the same group *P* < 0.01. Values are represented as mean ± sd. *P*-values^*a*^ were obtained using the Repeated analyses of covariance.*

No abnormalities were found in the blood routine, liver function, renal function, and electrocardiogram examinations after 8 weeks of treatment. TESS was used to assess the adverse reactions and influence of pharmacogenomic testing for medication tolerability. The incidence rate of adverse reactions was 55.56% in the guided group and 57.89% in the unguided group, respectively. However, no statistical difference was found between the two groups. The reported frequency of adverse reactions in the guided and unguided groups were 14 and 19 cases, respectively. The main adverse reactions were mild and tolerable symptoms, such as headache, dizziness, drowsiness, nausea, vomiting, dry mouth, constipation, diarrhea, decreased appetite, and tachycardia.

### Difference in the Outcome of Medication Classifications

The guided group had more patients in the “use as directed” category than the unguided group. Only 3.2% (1 case) of the guided group was in the “use with caution” category, whereas no patient was in the “use with increased caution and more frequent monitoring” category. Among the patients in the unguided group, 37.5% (15 cases) were in the “use as directed” category, 40% (16 cases) were in the “use with caution” category, and 22.5% (9 cases) were in the “use with increased caution and more frequent monitoring” category.

## Discussion

To our knowledge, this prospective single-blind study is the first to evaluate whether pharmacogenomic-guided treatment is more effective than unguided treatment in improving clinical outcomes for depressed patients in a Chinese population. The findings were inconsistent with our hypothesis and other previous studies, indicating that the pharmacogenomic-guided group had greater improvement in depressive symptom compared with the unguided group. Hall-Flavin et al. first evaluated the potential change in clinical outcomes by utilizing GeneSight testing that measures allelic variation among five genes (CYP2D6, CYP2C19, CYP1A2, SLC6A4, and HTR2A) in a small sample size of patients with MDD. They found a greater reduction of depression scores in the guided group compared with the unguided group at week 8 [30.8% vs. 18.2% in HAM-D17 and 31.2% vs. 7.2% in the Quick Inventory of Depressive Symptomatology–Clinician Rated (QIDS-C16)], and no statistically significant difference was found at weeks 2 and 4 (Hall-Flavin et al., 2012). The method without a double-blind design is similar to the one in the present study. Accordingly, Hall–Flavin et al. conducted a similar study with a larger sample size (72 and 93 cases in the guided and unguided groups, respectively) than their previous study and found that the clinical response rate was higher in the guided group (44.4%) than in the unguided group (23.7%) after an 8-week treatment (Hall-Flavin et al., 2013). A recent study using the GeneSight testing in measuring eight genes to assist in medication selection found that subjects with moderate-to-severe depression had a 27.9% of depression symptom reduction after 8–12 weeks; (Tanner et al., 2018), however, this study did not include a control group. By contrast, Winner et al. (2013) conducted a randomized double-blind study measuring five genes and showed that the guided group did not experience an improvement in HAMD-17 scores from the baseline compared with the unguided group (30.8% vs. 20.7%, *P* = 0.28). The work of Winner et al. was in line with the present study.

Several factors deserve consideration in explaining the negative result of this study. First, racial differences exist in the distribution of genetic variability for drug metabolizing enzymes in different populations. For example, the allelic frequency of CYP2D6^∗^10 is found in approximately 30%–50% of the Chinese population. This allelic frequency may influence the metabolism of psychotropic drugs and mostly presents as intermediate metabolizers; by contrast, ultrarapid metabolizers are rare (Hicks et al., 2013). Meta-analysis showed that CYP2C19^∗^2 loss-of-function variant is highly prevalent in Asian populations (78.5% of all variant alleles in East Asians). Among those of the genes analyzed, the allele frequency of CYP2C19^∗^17 ranges from 1.5% in East Asians to 22.4% and 23.5% in Europeans and Africans, respectively (Zhou et al., 2017). Second, PPS analysis found that nearly 37% (10 cases) of patients in the unguided group received drugs with the direct recommendation of the test report at the end of the study. This phenomenon may have affected the results. The doctor’s clinical experience also plays an important role. Third, many receptors, not just the inclusion of drug metabolism enzymes and SLC6A4 and 5-HTR2A genes, are involved in the efficacy of antidepressants. Hence, the detection sites must be increased to improve the accuracy of medication guidance.

Combinatorial pharmacogenomic testing from other platforms has also been reported. A multi-center, double-blind randomized study utilizing Neuropharmagen^®^, a commercial pharmacogenomic platform, developed by AB Biotics (Barcelona, Spain), showed higher clinical response rate in the guided group than in the unguided group (47.8% vs. 36.1%, *P* = 0.0476); the burden of side effects was also substantially reduced (Perez et al., 2017). However, the present work showed no statistical difference in the incidence rate of adverse reactions between the two groups. Brennan et al. utilized the Genecept assay (Genomind, King of Prussia, PA, United States), which was commercially available to guide prescription by evaluating allelic variations, including CYP2C19, CYP2D6, CYP3A4, 5HT2C, SLC6A4, dopamine-2 receptor, ankyrin g, L-type voltage-gated calcium channel, methylenetetrahydrofolate reductase, and catechol-*O*-methyltransferase. They found that 77% of the participants displayed improvement, 39% exhibited treatment response, and 38% reached full remission in a sample with 685 participants; (Brennan et al., 2015), however, their study has no control group. Another double-blind 12-week study utilized a proprietary pharmacokinetic interpretive report (CNSDose, developed by Baycrest Biotechnology Pty, Ltd.) that evaluates CYP2D6, CYP2C19, ABCC1, and ABCB1 transporter polymorphisms to help in medication dosing. Patients receiving guided prescriptions showed 72% remission, whereas the unguided group showed a remission rate of 28% (Singh, 2015). Altar et al. (2015a) combined the results of three clinical studies and found that pharmacogenomic-guided antidepressant therapy increases the response rate of treatment and alleviates depressive symptoms. However, a systematic review showed no significant difference in the rate of complete remission of depression between guided and unguided groups (Ontario, 2017).

Several limitations need to be considered in interpreting the results of this study. First, the sample size was small and did not include a representative enough sample of the population. The therapeutic effect of pharmacogenomic-guided treatment was possibly minimal. Similarly, the lack of a control group could distort the interpretation of the data. Age might have a confounding effect on depression and the patients’ response to the treatment. We have reanalyzed the data of HAMD-17 and HAMA using repeated analyses of covariance with age and gender as the covariates, and obtained similar results, suggesting that age and gender might have little effect on our findings. The dropout rate was high because some patients withdrew their consent or failed to follow up. Second, the study was not a multicenter report. The results may be biased because the patients who participated may not accurately represent the population with depression. Third, the study was not a double-blind trial. The placebo effect may have affected the results because the patients in the guided group knew about their genetic test reports prior to treatment. Fully blinding a combinatorial pharmacogenomic test has its own difficulties. By design, the information is multifactorial and therefore difficult to automate in a blinded fashion. Although full blinding in pharmacogenomics is a difficult task, it would give important and novel results. Therefore, a future full-blind clinical study with a large sample size and long follow-up time is recommended to examine the efficiency of pharmacogenomic testing.

## Conclusion

This study reports no significant difference in the improvement of depressive symptoms between guided and unguided groups at the end of the treatment. Pharmacogenomic testing might not significantly improve the clinical efficiency and safety for the guided group compared with those for the unguided group. Future full-blind randomization clinical study with a large sample size and long follow-up time is recommended to examine the efficiency of pharmacogenomic testing.

## Data Availability

All datasets generated for this study are included in the manuscript and/or the supplementary files.

## Ethics Statement

The Ethics Committee of the Second Xiangya Hospital of Central South University approved this study. After a complete explanation, all participants submitted their written informed consent.

## Author Contributions

LL, WG, JC, YF, and XS designed the study. XS, WZ, YQ, and HW collected the original data. XS wrote the first draft of the manuscript.

## Conflict of Interest Statement

The authors declare that the research was conducted in the absence of any commercial or financial relationships that could be construed as a potential conflict of interest.
